# The dual roles of chemokines in peripheral nerve injury and repair

**DOI:** 10.1186/s41232-025-00375-4

**Published:** 2025-04-11

**Authors:** Fangyuan Wang, Chenglin Zhao, Zhou Jing, Qingyi Wang, Minghe Li, Bingqi Lu, Ao Huo, Wulong Liang, Weihua Hu, Xudong Fu

**Affiliations:** 1 Department of Neurosurgery, The Fifth Affiliated Hospital of Zhengzhou University, Zhengzhou University, Zhengzhou, China; 2 Department of Neurosurgery, People’s Hospital of Zhengzhou University, Zhengzhou University, Zhengzhou, China; 3 Henan Provincial Key Laboratory of Cranial Nerve Diseases, ZhengZhou, China

**Keywords:** Chemokines, Peripheral nerve injuries, Inflammation

## Abstract

Peripheral nerve injuries (PNI) occur in approximately 13–23 per 100,000 individuals, predominantly affecting young and middle-aged adults. These injuries often require a lengthy recovery period, placing substantial burdens on healthcare systems and national economies. Current treatment strategies have not significantly shortened this lengthy regenerative process, highlighting the urgent need for innovative therapeutic interventions. Chemokines were originally noted for their powerful ability to recruit immune cells; however, as research has advanced, it has become increasingly evident that their role in peripheral nerve repair has been underestimated. In this review, we provide the first comprehensive overview of chemokine expression and activity during peripheral nerve injury and regeneration. We summarize the existing literature on chemokine family members, detailing their expression patterns and localization in injured nerves to facilitate further mechanistic investigations. For chemokines that remain controversial, such as CXCL1 and CCL2, we critically examine experimental methodologies and discuss factors underlying conflicting results, ultimately affirming their contributions to promoting nerve repair. Importantly, we highlight the dual nature of chemokines: in the early stages of injury, they initiate reparative responses, activate Schwann cells, regulate Wallerian degeneration, and support nerve recovery; but when the axons are connected and the repair enters the later stages, their persistent proinflammatory effects during later stages may impede the healing process. Additionally, we emphasize that certain chemokines, including CXCL5, CXCL12, and CCL2, can act directly on neurons/axons, thereby accelerating axonal regeneration. Future research should focus on precisely mapping the localization and temporal expression profiles of these chemokines and exploring therapeutic approaches.

## Introduction

Peripheral nerve injuries (PNI) affect between 13 and 23 per 100,000 individuals [1–4], predominantly occurring in the young and middle-aged population, with an average age of onset of 39.9 years [5]. Despite the availability of various treatment strategies—including direct surgical repair [6], nerve grafting [7], nerve conduits, photochemical tissue bonding [8], electrical stimulation (ES) [9], neurotrophic factors [10], and stem cell therapy [11]—recovery from PNI remains prolonged, and many patients are unable to achieve complete recovery throughout their lives. Furthermore, because these injuries primarily impact individuals of working age, they impose a substantial burden on both healthcare and socioeconomic systems [5]. Therefore, in-depth research into the mechanisms of PNI and its recovery methods is crucial for alleviating its burden on individuals and society.

The peripheral nervous system (PNS) consists of nerves outside the central nervous system (CNS) and differs significantly from the CNS in terms of structure and function. In the CNS, oligodendrocytes form axonal myelin sheaths [12], whereas Schwann cells perform this role in the PNS [13]. Notably, the PNS has a greater regenerative capacity than the CNS does, largely because of its unique biological properties. Schwann cells undergo rapid reprogramming and proliferation after injury, secreting proregenerative factors that reshape the microenvironment and form bands of Büngner to guide axonal regrowth [14]. Additionally, the relatively open immune system of the PNS allows immune cells to actively participate in repair processes. In contrast, CNS regeneration is limited by the restrictive blood‒brain barrier and the inhibitory local microenvironment [15]. Thus, understanding PNS repair mechanisms requires examining both the roles of neurons and the contributions of the repair microenvironment.

Peripheral nerve repair involves a complex interplay of molecular and cellular events, and the coordination of neurons, Schwann cells, and immune cells is critical. Following axonal injury, Schwann cells in the distal stump are reprogrammed into repair Schwann cells (rSCs), which perform multiple functions: they clear debris, recruit macrophages for Wallerian degeneration, align to form bands of Büngner that guide axonal regrowth, and secrete neurotrophic factors such as BDNF and NGF [16–18]. Once regenerating axons reach the injury site, rSCs transition back to myelinating Schwann cells to restore axonal function. Immune cells, particularly macrophages, also play vital roles. M1 macrophages facilitate debris clearance and inflammation, whereas M2 macrophages promote tissue regeneration through the production of reparative factors [19]. Other immune cells, such as T cells and neutrophils, modulate inflammation and support repair [20, 21]. Because various stem cells and immune cells are recruited to the injury site during the peripheral nerve repair process, especially in the early stages of healing, chemokines have long attracted the attention of researchers.

Chemokines, a class of cytokines with chemotactic activity, regulate immune cell migration, localization, and function [22]. They are categorized into four subfamilies—CXC, CC, CX3 C, and XC—based on the arrangement of cysteine residues at their N-terminus [23]. Early research in the field of chemokines typically focused on immune regulation, viewing them solely as tools for recruiting immune cells to damaged tissues. However, in recent years, increasing evidence has demonstrated that chemokines can directly act on neurons and glial cells to promote axonal regeneration. This suggests that chemokines have significant potential roles in nerve repair. Unfortunately, to date, no scholars have provided a systematic summary of the expression patterns and mechanisms of chemokines in peripheral nerves, leaving researchers without comprehensive references when conducting studies in this area. Therefore, we have reviewed the expression patterns and mechanisms of the chemokine family in PNI and presented our perspectives on some controversial viewpoints to facilitate and support future research in this field. Additionally, we were pleasantly surprised to find that the roles of the chemokine family closely align with the early stages of injury repair. However, once severed axons reconnect and the nerve repair process enters its later stages, an excessive inflammatory response may inhibit remyelination by Schwann cells and the shift of macrophages toward an anti-inflammatory phenotype, ultimately leading to unfavorable outcomes. This finding suggests that the early phase of repair may be a critical window for the action of the chemokine family.

## Expression and localization of chemokine families

The expression and localization of chemokines and their receptors are critical for elucidating the mechanisms by which chemokine families’ function in PNI and for exploring their potential roles. Table 1 summarizes the expression and localization of chemokines and their receptors in the context of PNI, showing their distribution across different cell types and different time points post-injury. However, nearly all studies on the injury microenvironment are based on animal models, and to date, no studies have reported changes in the injury microenvironment following human nerve injury. Although this limitation is understandable owing to practical challenges, such as ethical restrictions and limited sample availability, it remains a critical gap in our understanding. In light of this challenge, we propose a potential solution: because harvesting nerve tissue itself causes additional harm to the patient and conflicts with the goal of nerve repair, it should not be performed during standard procedures such as nerve anastomosis or transplantation. However, for patients requiring amputation, it may be possible—pending ethical approval and patient consent—to collect healthy or injured nerve tissue. This approach could help overcome the current lack of human-based studies in this field. Investigating the expression levels and temporal dynamics of chemokines is of critical importance for identifying potential downstream pathways, exploring their functional roles, and informing intervention strategies. In PNI, the expression of chemokines and their receptors is influenced by various factors, including time post-injury, immune cell infiltration, injury type and severity, nerve location, and age. Overall, neurons/axons and Schwann cells are important sources of chemokines, and the expression of many chemokines is rapidly upregulated in the early stages following PNI. This upregulation is significantly associated with the recruitment of various immune cells and potentially stem cells during the early phase of nerve injury, which aligns with the traditional view that chemokines play a key role in the recruitment of immune cells during the initial stages of injury. However, certain chemokines, such as CXCL10, are predominantly expressed at later stages of nerve repair. By this point, severed axons have already reconnected, and the elevated levels of CXCL10 bind to the CXCR3 receptor on macrophages, promoting their recruitment and inflammatory polarization. Consequently, these chemokines do not facilitate early clearance of necrotic tissue or the establishment of a regenerative environment; instead, their late-stage expression may contribute to excessive inflammation, ultimately hindering nerve recovery.

**Table 1:** Expression and localization of chemokines in different families

	Localization	Summary of expression changes	Model	Reference
C-X-C chemokine subfamily				
CXCR2	Neutrophils		SNT	[24]
	Neurons/axons	Transcript levels in the DRG increase immediately post-injury, peaking on day 1; protein levels significantly rise by day 3	CFA	[25]
	Macrophages	Protein levels in the injured tissue gradually increase over the first 3 days post-injury, peaking on day 3	SNC	[26]
CXCR3	Macrophages		SNC SNT	[27]
CXCR4	Neurons/axons	Protein levels in the injured tissue increase post-injury	SNC	[28]
	Schwann cells	Protein levels in the tissue significantly decrease on the second day after nerve injury, gradually recover, and decrease again about 3 weeks post-injury	SNC	[29]
	CD34^+^ cells			[30]
	ADSCs			[31]
	Mesenchymal stem cells			[32]
CXCL1	Neurons/axons	Transcript levels in the DRG increase immediately after injury, peaking on day 1, with protein levels significantly elevated on days 1 and 3	CFA	[25]
	Schwann cells	Transcript levels in the injured tissue rise immediately post-injury and then decrease; transcript levels on day 3 are much lower than those on day 1	SNT	[33]
	Schwann cells	Protein levels in the injured tissue significantly increase within 24 h post-injury	SNC	[26]
CXCL2		Protein levels are increased on days 1 and 14 post-injury	SNT	[34]
		No CXCL2 protein expression is detected from day 0 to day 30 post-injury	Tellurium SNC SNT	[35]
CXCL3		Protein levels increase on day 1 post-injury in the injured tissue	SNT	[34]
CXCL5	Neurons/axons	Protein levels increase by 1.5-fold in the SCG and 1.8-fold in the DRG at 48 h post-injury	ECNT ICNT SNT	[36]
CXCL6		Protein levels increase on day 1 post-injury in the injured tissue	SNT	[34]
CXCL9		Protein levels increase on day 1 post-injury in the injured tissue	SNT	[34]
CXCL10		Protein levels increase on days 3 and 7 post-injury in the injured tissue	SNC	[37]
CXCL11		Protein levels increase on day 1 post-injury in the injured tissue	SNT	[34]
CXCL12	Neurons/axons	Transcript levels increase in the MPG on day 1 post-injury	CNC	[38]
	Schwann cells	No CXCL12 expression is detected in normal nerve tissue Protein levels increase in the injured tissue on days 3 and 7 post-injury but nearly return to normal by day 7	SNC	[28]
		Protein levels in injured tissue show a strong but brief increase on day 1, decrease by day 14, and return to normal by day 28	FNC	[39]
		Protein levels increase on day 7 post-injury in the injured tissue	CNC	[40]
		Protein levels peak between days 10 and 14 post-injury in the injured tissue	SNC	[41]
CXCL13	Neurons/axons	Transcript levels in the DRG increase between days 3 and 14 post-SNL	L4 SNL	[42]
		Protein levels increase on day 1 post-injury in the injured tissue	SNT	[34]
C–C chemokine subfamily				
CCR2	Macrophages		SNT	[43]
CCR4	Macrophages		SNT	[44]
	ADSCs		CNC	[38]
CCL1		Protein levels increase on day 14 post-injury in the injured tissue	SNT	[34]
CCL2	Schwann cells (primary source), neurons/axons, macrophages, fibroblasts	The number of CCL2^+^ cells increases at 6 h post-injury, continues to rise, and peaks between 2 and 4 weeks	SNT	[45]
		Protein levels increase on days 1 and 14 post-injury in the injured tissue	SNT	[34]
		Transcript levels in the distal injured tissue peak on day 1 post-injury, decrease on days 3 to 7, and rise to a second peak on day 14. Levels return to normal between days 21 and 28. In proximal tissue, CCL2 transcript levels remain elevated on day 28	SNT	[46]
		Transcript and protein levels in the injured tissue remain elevated for 21 days post-injury compared to normal tissue	L5 transection	[47]
	Neurons/axons	Transcript levels increase in the MPG on day 1 post-injury	CNC	[38]
	Macrophages	Both protein and transcript levels increase in macrophages post-injury; however, protein levels in repeatedly injured tissue are significantly lower than those in tissues that experience a single injury	SNC	[48]
		At 24 h post-injury, CCL2 transcript levels in the nerves in young mice are higher than those in in older mice	SNT	[49]
	Neurons/axons	Transcript levels in the L5 DRG increase 2.9-fold on day 7 post-sciatic nerve injury	SNT	[50]
	Schwann cells Macrophages	Transcript levels in Schwann cells are six times higher than those in macrophages on day 14 post-injury	SNT	[43]
	Neurons/axons	Transcript levels peak at 48 h post-injury in the pelvic ganglia after cavernous nerve crush injury	CNC	[51]
		Transcript levels rise within 12 h post-injury, peaking between days 1 and 3 in the injured tissue	SNT	[33]
	Neurons/axons	Transcript levels increase in the DRG between days 1 and 16 post-injury	SNT	[52]
		Transcript levels peak on day 3 post-injury in the injured tissue	Tellurium SNC SNT	[35]
CCL3		Transcript levels in the distal injured tissue peak on day 1, decrease on days 3 and 7, and rise to a second peak on day 14, returning to normal levels between days 21 and 28	SNT	[46]
		Protein levels increase on day 1 post-injury in the injured tissue	SNT	[34]
	Schwann cells Neurons/axons Macrophages Fibroblasts	The number of CCL3^+^ cells increase at 24 h post-injury, remains stable for over 4 weeks, and peaks on day 5	SNT	[45]
		Transcript and protein levels in the injured tissue remain elevated for 21 days post-injury compared to those in normal tissue	L5 transection	[47]
CCL5	Schwann cells Neurons/axons Macrophages Fibroblasts	The number of CCL5^+^ cells increase at 24 h post-injury, remains stable for over 4 weeks, and peaks on day 3	SNT	[45]
		Transcript levels in the distal injured tissue peak on day 1, decrease on days 3 and 7, rise to a second peak on day 14, and return to normal between days 21 and 28 Proximal transcript levels show minimal changes on day 1 and return to control levels by day 3	SNT	[46]
		Transcript and protein levels in the injured tissue remain elevated for 21 days post-injury compared to those in normal tissue	L5 transection	[47]
		Protein levels increase on day 14 post-injury in the injured tissue	SNT	[34]
CCL7		Protein levels increase on days 1 and 14 post-injury in the injured tissue	SNT	[34]
CCL15		Protein levels increase on day 14 post-injury in the injured tissue	SNT	[34]
CCL17		Protein levels increase on day 1 post-injury in the injured tissue	SNT	[34]
C-X3-C chemokine subfamily				
CX3 CR1	ADSCs		CNC	[38]
CX3 CL1	Neurons/axons	Transcript levels increase in the MPG on day 1 post-injury	CNC	[38]
X-C chemokine subfamily				
XCR1	ADSCs		CNC	[38]
	Neurons/axons CD45^+^ WBC Schwann cells	Protein levels increase on days 1 and 3 post-injury, and XCR1 transcript levels increase on day 3 post-injury in the injured tissue	CCI	[53]
XCL1	Neurons/axons	Transcript levels increase in the MPG on day 1 post-injury	CNC	[38]

*SNT* sciatic nerve transection, *SNC* sciatic nerve crush, *FNC* facial nerve crush, *CNC* cavernous nerve crush, *CCI* chronic constriction injury, *SNL* sciatic nerve ligation, *ADSCs* adipose-derived stem cells, *ECNT* external carotid nerve transection, *ICNT* internal carotid nerve transection, *MPG* major pelvic ganglion

Furthermore, it is important to note that some studies have only investigated protein levels in upstream ganglia or neurons in the injured nerve, but these data may not accurately reflect changes in protein levels within the local microenvironment at the site of injury. Protein expression patterns in neurons, such as those in the dorsal root ganglion (DRG), can differ significantly from those at the local injury site, such as the sciatic nerve. Local cells, including Schwann cells and infiltrating immune cells, have a major impact on protein expression within the injury microenvironment. Additionally, although retrograde axonal transport conveys injury signals to neuronal cell bodies to initiate transcriptional and translational responses, the resulting proteins are not always transported back to the injury site.

**Fig. 1:**
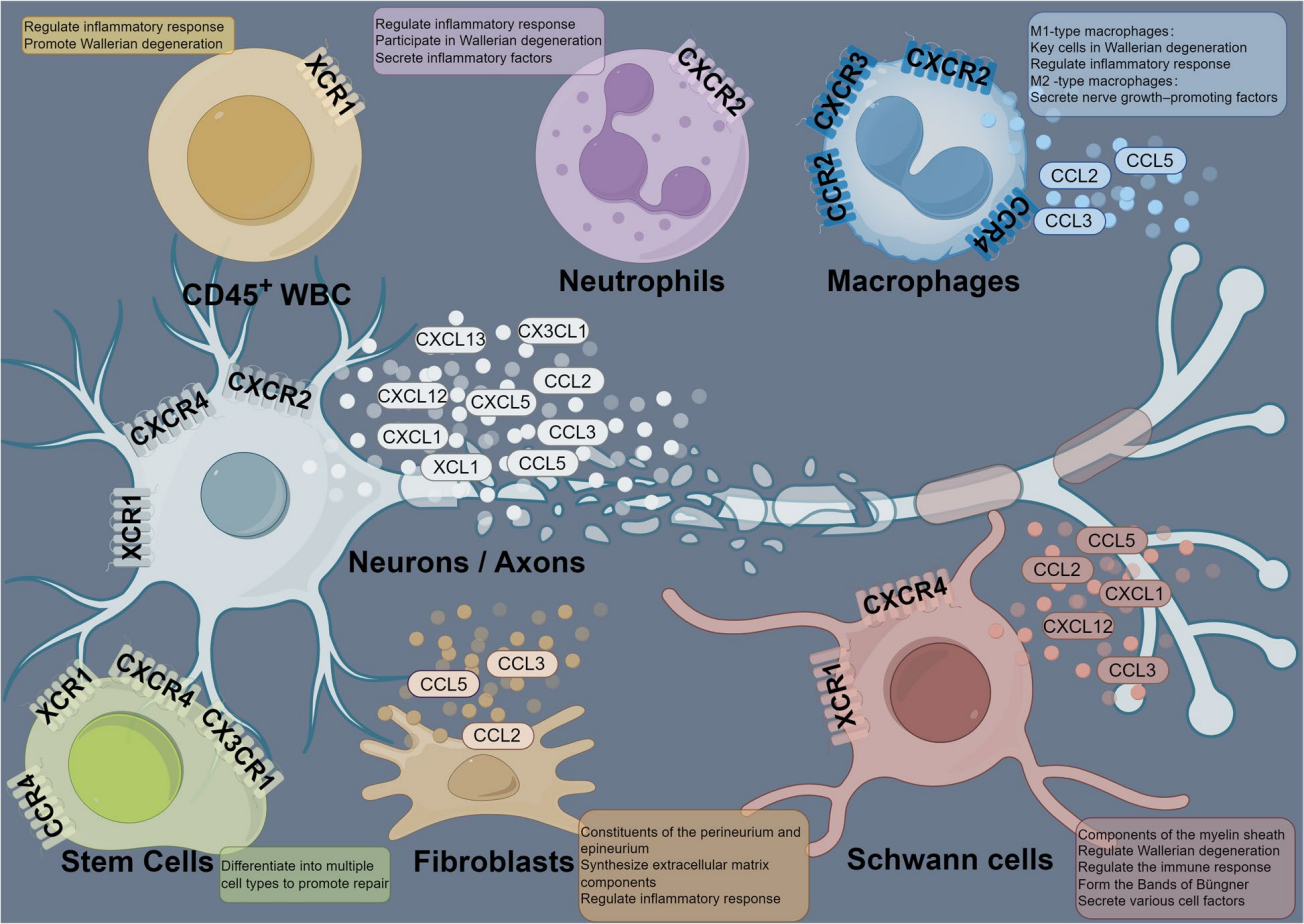
Localization of the chemokine family. Neutrophils express CXCR2. Macrophages express CXCR2, CXCR3, CCR2, and CCR4 on the cell surface and secrete CCL2, CCL3, and CCL5. Neurons/axons express CXCR2, CXCR4, and XCR1 on the cell surface and secrete CXCL1, CXCL5, CXCL12, CXCL13, CCL2, CCL3, CCL5, CX3 CL1, and XCL1. Schwann cells express CXCR4 and XCR1 on the cell surface and secrete CXCL1, CXCL12, CCL2, CCL3, and CCL5. Adipose-derived stem cells (ADSCs) express CXCR4, CCR4, CX3 CR1, and XCR1 on the cell surface. CD34⁺ cells and mesenchymal stem cells express CXCR4 on the cell surface. CD45⁺ WBC express XCR1 on the cell surface. Fibroblasts secrete CCL2, CCL3, and CCL5

The localization of chemokines and their receptors is also of substantial importance. Specifically, understanding receptor localization helps identify chemokine targets, thereby guiding subsequent research toward examining their effects on particular cell types. Unfortunately, studies in this area are notably limited. Most existing work focuses solely on the overall expression of chemokines within injured tissue, without addressing their precise cellular localization. This knowledge gap complicates our efforts to elucidate the exact mechanisms governing chemokine expression and to characterize their cellular effects following activation. To assist future researchers, we present the localization of chemokine family members in Fig. 1. The available findings indicate that chemokine receptors are widely distributed across injured tissues, including neurons/axons, glial cells, immune cells, and stem cells. This broad distribution underscores the critical role of chemokine/receptor signaling in nerve injury and repair, highlighting the potential of chemokines as therapeutic targets. Chemokine ligands are derived primarily from Schwann cells and neurons/axons. However, as previously mentioned, whether chemokines secreted by neuronal cell bodies can be transported through axons to the injury site to exert their effects requires further investigation.

## The C-X-C chemokine subfamily

The CXC chemokine family is a chemokine subfamily named for the presence of one amino acid (X) between two N-terminal cysteine residues. CXC chemokines play significant roles in inflammatory responses, immune regulation, tumor development, and nerve injury and repair processes. The CXC chemokine subfamily comprises seven receptors (CXCR1–CXCR7) and 17 ligands (CXCL1–CXCL17), among which CXCL1, CXCL5, and CXCL12 play important roles in nerve repair.

### CXCL1/CXCR2 may exert neuroprotective effects via a neutrophil-dependent mechanism

Currently, the role of CXCL1/CXCR2 in nerve repair remains controversial. However, we tend to view the CXCL1/CXCR2 axis as a neutrophil-dependent neuroprotective pathway. Studies have shown that recombinant CXCL1 can exert neuroprotective effects by mediating neutrophil infiltration [47]. Additionally, elevated reactive oxygen species (ROS) levels in damaged muscle can recruit CXCR2-positive neutrophils to indirectly injured muscle tissue via a CXCL1-dependent pathway, thereby delaying muscle atrophy [24].

However, it is worth noting that a study by Jiang et al. proposed a different viewpoint. Their findings suggested that CXCL1 promotes macrophage migration by binding to CXCR2 on the macrophage surface. This interaction not only accelerates macrophage recruitment but also activates inflammatory pathways (such as the NLRP3 inflammasome pathway), inducing the production of proinflammatory cytokines such as IL- 1β and thereby inhibiting functional recovery after sciatic nerve injury. Importantly, that study mainly relied on the application of the CXCR2 antagonist SB225002 rather than the use of specific CXCL1 inhibitors, CXCL1 knockout models, or recombinant CXCL1 protein injections to verify its conclusions. This experimental design may be insufficient to accurately define the role of CXCL1. Furthermore, CXCL1 protein expression significantly increased within 24 h after nerve injury but rapidly decreased after 72 h. These findings suggest that the time window for the action of CXCL1 may be concentrated during the early stages after injury, especially on the first day. Clearly, the long-term use of SB225002 by Jiang et al. differs from the expression pattern of CXCL1 [26].Thus, this administration approach does not align with the expression profile of CXCL1. On the basis of these findings, the role of the CXCL1–CXCR2–NLRP3–IL- 1β pathway in nerve repair requires further exploration, and the CXCL1/CXCR2 axis is more likely to be a protective pathway.

In summary, after a thorough analysis of these two opposing perspectives, we conclude that the CXCL1/CXCR2 axis exhibits a neuroprotective role through neutrophil-mediated mechanisms. However, further research using more precise experimental approaches is crucial to address current controversies and comprehensively clarify its role in nerve repair.

### CXCL5/CXCR2 induces neuronal sensitization but promotes nerve repair

PNI often leads to increased neuronal responsiveness to stimuli, resulting in exaggerated reactions to external stimuli or strong responses to minor ones—a condition known as neuronal sensitization. This phenomenon is particularly prominent in pain perception and plays a critical role in the development of chronic pain. Consequently, many animal models of chronic inflammation are established by inducing peripheral nerve damage. However, studies have shown that neuronal sensitization and nerve regeneration are two independent events. For example, Zhu et al. reported that although CCL2 can induce nociceptive sensitization, it simultaneously promotes the regeneration of dorsal root ganglion (DRG) neurons [54]. Similarly, CXCL5 may play dual roles in neuropathic pain induction and nerve repair.

Previous studies have generally recognized the role of CXCL5 in nerve repair. For example, CXCL5 secreted by adipose-derived stem cells can promote neurite outgrowth in major pelvic ganglion (MPG) neurons and activate the JAK/STAT pathway in Schwann cells, promoting cavernous nerve regeneration [55]. Platelet-rich plasma (PRP) contains high levels of CXCL5; its injection into the corpus cavernosum can stabilize CXCR2 and increase CXCL5 expression in the MPG in a bilateral cavernous nerve crush (BCNC) model, thereby enhancing nerve repair capacity [56]. Low-energy defocused shock wave (DLSW) therapy increases the secretion of CXCL5 and VEGF from bone marrow-derived mesenchymal stromal cells (BMSCs), increasing the neurite outgrowth ability of pelvic ganglion neurons [57]. Interestingly, CXCL5 has also been defined as a protective factor in the context of injury to the optic nerve, which is part of the central nervous system. Although the optic nerve belongs to the central nervous system, the molecular pathways and cellular responses involved in axon regeneration are similar in both central and peripheral neurons/axons. Liu et al. reported that recombinant CXCL5 can promote neurite growth in retinal ganglion cells (RGCs) in retinal explants and facilitate axon regeneration after optic nerve crush injury by activating the Akt and STAT3 signaling pathways [58]. These molecular pathways and cellular responses may similarly occur in neurons and axons within the PNI environment, where they could play a direct role in neuroprotection and axonal regeneration. This warrants further investigation.

Moreover, the ability of CXCL5 to mediate neuronal hypersensitivity is also worth noting. Xu et al. reported that the intrathecal injection of CXCL5 can induce nociceptive hypersensitivity and is associated with neuropathic pain in spinal dorsal horn neurons in a rat model of chronic constriction injury (CCI) [59]. Overall, CXCL5 may play dual roles in neuropathic pain induction and nerve repair. Given that research on the role of CXCL5 in nerve repair is relatively limited and that the expression patterns and localization of CXCL5 remain unclear, future studies should explore this topic in depth to verify the nerve regeneration effects and specific mechanisms of CXCL5.

### CXCL12/CXCR4: a key axis in nerve repair

CXCL12, the only high-affinity endogenous ligand for CXCR4, is widely regarded as beneficial for nerve repair. CXCR4 agonists such as NUCC- 390 enhance neuromuscular recovery, axonal elongation, and repair efficiency [28]. This axis directly exerts neurotrophic effects by increasing neurofilament light chain (NF-L) expression, neuronal differentiation, and axonal growth [60, 61]. CXCL12 at the injury site acts on neural progenitor cells via CXCR4, regulating migration speed through the ERK1/2 and p38 MAPK pathways and directionality via the Akt and JNK pathways[40, 62–64]. Additionally, CXCL12 indirectly facilitates repair by recruiting multiple cell types through the widespread expression of CXCR4. For example, CXCL12 promotes Schwann cell migration and autophagy through the PI3 K/AKT/mTOR pathway without affecting proliferation or apoptosis[39]. It also enhances the migration of CD34 + hematopoietic and adipose-derived stem cells (ADSCs) [30, 31]. Zhang et al. reported that the overexpression of Neuritin increased CXCL12 levels in sciatic nerve tissue and regulated the migration of bone marrow-derived mesenchymal stem cells (MSCs) through the CXCL12/CXCR4-PI3 K/Akt signaling pathway, thereby promoting nerve repair [32].

Innovative drug delivery systems utilizing CXCL12 have shown promise; for example, Jian et al. employed a glycosaminoglycan-based hybrid hydrogel with encapsulated polyelectrolyte complex nanoparticles (PCNs) based on sulfated glycosaminoglycans as a delivery platform for CXCL12 and bFGF. This hydrogel facilitated the recruitment of endogenous neural stem cells (NSCs) through CXCL12 signaling [65]. Additionally, incorporating CXCL12 and bFGF into nerve conduits significantly enhanced the recruitment of CD34 + cells and promoted angiogenesis, thereby effectively accelerating nerve regeneration [66]. Overall, the CXCL12–CXCR4 axis is a robust therapeutic target for nerve repair.

### Roles of additional C-X-C family members in peripheral nerve repair

In addition to the ligands of CXCR2 and CXCR4, other CXC chemokine subfamily members, such as CXCL10 and CXCL13, also play pivotal roles in peripheral nerve repair.

The expression pattern of CXCL10, the specific ligand of CXCR3, is tightly correlated with that of CXCR3. Notably, CXCR3 knockout significantly reduces CXCL10 expression in tissues. Research has indicated that CXCL10 expression begins on day 3 after nerve injury and progressively increases over time, although the precise localization of CXCL10 remains to be fully elucidated. Functionally, the knockout of CXCR3 significantly reduces macrophage recruitment, and CXCR3-deficient mice exhibit enhanced nerve repair capabilities [37]. Interestingly, CXCL10 expression begins around day 3 post-injury and remains elevated during the later stages of repair. Based on this delayed expression pattern, we hypothesize that macrophages recruited by CXCL10 may not sufficiently participate in early Wallerian degeneration but instead contribute to excessive inflammation during the late phase of repair. Could modulating the timing of CXCL10-CXCR3 activation—inducing rapid activation in the early phase and suppressing it in the late phase—offer better therapeutic outcomes compared to full suppression of CXCR3 throughout the repair process?

CXCL13, which binds both CXCR3 and CXCR5 [67, 68], is secreted by the DRG and has a unique regulatory mechanism following nerve injury. ZNF382a is a nuclear transcription factor that interacts with the distal silencer region of the CXCL13 gene and forms a complex with HDAC1 and SETDB1 at the promoter region. This complex occupies the promoter and 5′-UTR regions of the CXCL13 gene, forming a silencer–promoter loop that suppresses CXCL13 transcription in normal DRG neurons. Following PNI, ZNF382 expression in the DRG decreases, leading to increased acetylation of histone H3 (ac-H3) in the F11 fragment and reduced H3 K9 me3 enrichment in the F10 fragment within the promoter and 5′-UTR regions of the CXCL13 gene. This results in the activation of CXCL13 transcription in injured DRG neurons. Functionally, CXCL13 binding to CXCR5 contributes to pain hypersensitivity, highlighting the role of CXCL13 in neuropathic pain [42].

In conclusion, CXCL10 and CXCL13 participate in peripheral nerve repair and pathology through immune cell recruitment and epigenetic regulation, respectively. These findings identify these two chemokines as promising therapeutic targets, providing new opportunities for improving nerve regeneration and managing neuropathic pain.

## The C–C chemokine subfamily

The CC chemokine subfamily comprises 10 receptors (CCR1–CCR10) and 28 ligands (CCL1–CCL28). Omics studies have revealed a significant increase in the expression of several CC family members, including CCL1, CCL2, CCL3, CCL5, CCL7, CCL15, and CCL17, after PNI. These chemokines are heavily involved in various nerve repair processes, such as macrophage recruitment and polarization, Wallerian degeneration, Schwann cell function regulation, and the promotion of nerve growth [34, 46, 69]. Among these chemokines, CCL2 and its receptor CCR2 play critical roles in nerve injury and repair. Although some studies have shown that CCL2 can act directly on neurons or axons, there is currently no definitive evidence showing the presence of CCR2 in cell types other than macrophages within the PNI microenvironment.

### TLR signaling, ER stress, and ion channels mediate CCL2 upregulation after nerve injury

The mechanisms underlying the increased expression of CCL2 after nerve injury have been widely investigated. Necrotic neurons or nerve tissue homogenates can activate Schwann cells through Toll-like receptors (TLR2, TLR3, and TLR4), promoting the expression of multiple genes, including CCL2 [70, 71]. Research has confirmed that TLR2 and TLR4 ligands enhance CCL2 expression via a MyD88-dependent pathway. S100 A8/A9 may also play an important role in this process [72]. The genes S100a8 and S100a9 are highly upregulated on the first day after nerve injury in mice, and the heterodimeric noncovalent S100 A8/A9 complex is directly associated with the upregulation of Ccl2, Ccl7, and Cxcl2 expression in Schwann cells [73].

Moreover, disruption of endoplasmic reticulum (ER) homeostasis contributes to increased expression of CCL2. Following PNS or CNS injury, ER protein homeostasis is disrupted, leading to protein-folding stress responses in neurons and glial cells. ER stress activates the unfolded protein response (UPR), which results in splicing of the Xbp1 mRNA by IRE1α, generating the active transcription factor XBP1 s. This, in turn, enhances CCL2 expression [74].

Voltage-gated ion channels are also involved in the regulation of CCL2 expression in neurons. For example, ropivacaine can reduce the expression levels of Nav1.8 in sciatic nerve axons (Nav1.8 is expressed mainly in substance P-positive peptidergic axons), thereby promoting the release of CCL2 from axons to recruit macrophages (primarily the M1 type). Additionally, ropivacaine can induce the polarization of M1 macrophages toward the M2 phenotype [75].

In summary, the upregulation of CCL2 following nerve injury is orchestrated by TLR signaling, ER stress responses, and voltage-gated ion channel activity, collectively contributing to the inflammatory milieu and influencing nerve repair processes.

### CCL2 promotes peripheral nerve repair via macrophage-dependent pathways and direct neurotrophic effects

CCL2 plays a pivotal role in nerve repair by acting as a crucial mediator that bridges various cellular processes that are highly dependent on the recruitment and polarization of macrophages. In the context of PNI, macrophage recruitment primarily depends on CCR2 expression [76]. As a CCL2 is a key factor that activates CCR2 and mediates macrophage recruitment, reduced expression of CCL2 significantly inhibits macrophage recruitment and impedes Wallerian degeneration. When CCL2 is knocked out, CCL7 and CCL12 can partially compensate for its absence, maintaining effective clearance of damaged tissues during the early stages of injury [77]. Macrophages activated via the CCL2/CCR2 axis polarize toward the M1 phenotype through signaling pathways such as the Jak-STAT and Ras pathways; they then secrete IL- 6 and IL- 1 to enhance the inflammatory response, promote angiogenesis at the injury site, and support neurite growth [43, 51, 69]. Furthermore, resident macrophages are critical for neurogenesis and maturation; depletion of macrophages leads to increased neuronal death and reduced neurogenesis in the olfactory epithelium of mice [78].

CCL2 can also directly promote neuronal regeneration through a STAT3-dependent mechanism. Studies have demonstrated that the overexpression of CCL2 selectively increases LIF mRNA levels and activates STAT3, which is essential for neurite growth. Inhibition of STAT3 phosphorylation eliminates these regenerative effects [50]. The neurotrophic effect of CCL2 has been validated in dorsal root ganglion (DRG) neurons, where it increases the expression of GAP43 and ATF3, significantly promoting nerve regeneration [54].

In summary, the CCR family—particularly the CCL2/CCR2 axis—plays multiple critical roles in nerve repair. It not only promotes nerve growth by directly acting on neurons and axons but also accelerates Wallerian degeneration and facilitates angiogenesis through the recruitment and polarization of macrophages. Therapeutic strategies targeting the CCL2/CCR2 axis have shown significant clinical potential. For example, overexpressing CCL2 in DRG neurons via viral vectors significantly promoted sensory nerve axon regeneration in a rat model of spinal cord injury [50]. Chitooligosaccharide (COS) downregulates miR- 327, preventing miR- 327 from post-transcriptionally downregulating CCL2 expression in Schwann cells by targeting its 3′-UTR [79]. Additionally, the serum-free conditioned medium secreted by stem cells from human exfoliated deciduous teeth (SHED-CM), when implanted in collagen sponges, effectively restores nerve function after facial nerve transection in rats; this functional recovery is significantly dependent on CCL2 and sialic acid-binding immunoglobulin-like lectin- 9 (sSiglec- 9) [80].

Nonetheless, it should not be overlooked that in some nontargeted CCL2/CCR2 therapeutic strategies (e.g., TET2 overexpression or hyperbaric oxygen therapy) [81, 82], nerve repair outcomes remain significantly improved despite decreased CCL2 expression. This phenomenon may be attributed to the dynamic and dual roles of inflammation in nerve repair. However, we still tend to view the activation of the CCL2/CCR2 axis as a neuroprotective factor because overexpressing CCL2 alone significantly promotes nerve repair, whereas knocking out CCR2 markedly inhibits the repair process. These findings underscore the critical and irreplaceable role of the CCL2/CCR2 axis in nerve injury repair.

## The C-X3-C chemokine subfamily

Currently, the CX3 C chemokine subfamily consists only of CX3 CR1 and its ligand CX3 CL1. Studies have shown that the CX3 C family plays a crucial role in the repair process after nerve injury by recruiting macrophages and adipose-derived stem cells (ADSCs).

CX3 CR1-positive macrophages are considered key factors in nerve repair. For example, in a sciatic nerve injury model, researchers reported that CX3 CR1-positive macrophages recruited to the site of injury release exosomes containing functional NADPH oxidase 2 (NOX2) complexes. Active NOX2 enters damaged nerve axons via endocytosis and is to the cell body through retrograde transport via an importin-β1–dynein–dependent mechanism. This process leads to the oxidation and inactivation of PTEN, thereby activating the PI3 K–Akt signaling pathway and promoting nerve regeneration [27]. Clinically, targeting the CX3 CR1–NOX2 axis may enhance nerve repair. Supporting CX3 CR1-positive macrophages could bolster NOX2 signaling, promoting axon regrowth via PTEN oxidation and PI3 K–Akt activation. However, excessive ROS scavenging may hinder this effect, so antioxidant timing and dosage require caution. Pharmacological modulation of PTEN or PI3 K–Akt might further aid recovery. Additional studies are needed to define safe, effective treatments for nerve injury patients. Similar findings have been validated in optic nerve (central nervous system) injury models. Investigators have reported that activation of the CX3 CL1‒CX3 CR1 axis promotes the expression of regeneration-associated proteins—such as βIII-tubulin, BRN3 A, and GAP43—in retinal ganglion cells (RGCs), thereby facilitating RGC regeneration [83]. Additionally, the CX3 CL1/CX3 CR1 signaling pathway may accelerate cavernous nerve repair by promoting homing of ADSCs to the site of injury [38].

Notably, other studies have demonstrated that the CX3 CL1/CX3 CR1 axis plays an important role in the proliferation, maturation, and dendrite development of newborn hippocampal neurons[84]. These findings suggest that CX3 CL1/CX3 CR1 may also directly promote peripheral nerve regeneration. Future research should further explore the specific mechanisms by which this axis exerts its effects in the context of different types of nerve injuries.

## The X-C chemokine subfamily

The XC chemokine subfamily is a unique subgroup within the chemokine superfamily, characterized by members containing only a single cysteine residue, which significantly differentiates their structure from that of members of other chemokine families. Known XC family members include XCL1 (lymphotactin-α) and XCL2 (lymphotactin-β), both of which exert their biological effects by binding to the specific receptor XCR1.

Studies have indicated that the XCL1-XCR1 axis may play an important role following nerve injury. For example, researchers have reported that XCL1 increases the excitability of trigeminal neurons by binding to XCR1, thereby mediating trigeminal neuralgia [53]. Similar findings were obtained in other studies, where XCL1 was observed not only to bind to XCR1 but also to activate ITGA9 (a transmembrane receptor protein belonging to the integrin family), leading to neuropathic pain after chronic constriction injury (CCI) [85]. Moreover, similar to the CX3 CL1/CX3 CR1 axis, XCL1 was found to be significantly upregulated in major pelvic ganglion (MPG) cells 24 h after cavernous nerve (CN) injury. XCL1 has been identified as a factor that may promote homing of adipose-derived stem cells (ADSCs) to the MPG. However, research on this topic remains quite limited [38]. Thus, the currently available findings are insufficient to definitively determine the specific role of XC family members in nerve repair. Additional studies are needed to elucidate the mechanisms and potential therapeutic applications of the XC chemokine subfamily in neural regeneration.

## Discussion

PNI have a substantial negative impact on society, and current treatment approaches still fail to significantly shorten the lengthy recovery period. In fact, some patients may never achieve full recovery [86, 87]. As a form of traumatic damage, inflammation plays an irreplaceable role in PNI. Chemokines, a classic family of inflammatory mediators, have long attracted attention for their powerful ability to recruit immune cells. However, recent research has led to a growing realization that their roles in peripheral nerve injury and regeneration have been underestimated. In this review, we provide the first comprehensive overview of the expression and functions of the chemokine family in nerve injury and repair. We summarize existing findings on chemokine expression patterns and localization, laying a foundation for future investigations into their potential mechanisms. For chemokines whose functions remain controversial, such as CXCL1 and CCL2, we scrutinize previous experimental methodologies to clarify the underlying reasons for these opposing viewpoints and confirm their protective roles in nerve injury. Additionally, we hypothesize that chemokines exhibit dual characteristics in nerve injury, with the timing of chemokine pathway activation likely determining whether their effects are beneficial or detrimental. Based on these insights, we propose that early administration of certain chemokines—such as CXCL5, CXCL12, and CCL2—that can directly act on neurons/axons may represent a promising therapeutic strategy to enhance peripheral nerve repair [50, 55, 61].

Inflammation is an indispensable part of the injury repair process, and chemokines, as classical inflammatory mediators, play a pivotal role in this context [88]. Recent advancements in transcriptomics and proteomics have revealed the critical regulatory functions of chemokines in peripheral nerve repair. However, to date, studies have predominantly focused on analyzing the transcript or protein levels of chemokines at the whole-tissue level, with insufficient exploration of the specific sources of chemokines and their receptors. Several key questions remain unanswered: In which cells are chemokine receptors expressed? Which cells are the primary sources of chemokines in injured tissues? Which cells secrete these chemokines? Answers to these questions are crucial for developing new treatments. Clarifying the localization of chemokine receptors can reveal potential targets of differentially expressed chemokines within tissues. Additionally, tracing the origins of neuroprotective chemokines can inform therapeutic strategies aimed at increasing chemokine protein levels in injured tissues via exosome technology or stem cell transplantation. We therefore look forward to future studies that will clarify the precise localization of chemokines and their receptors in injured tissues, and comprehensively elucidate the origins and fates of these chemokines throughout both the injury and repair processes.

It is also essential to distinguish between the expression of chemokines in specific cell types and the overall expression of chemokines in the injury microenvironment. PNI primarily occurs in the distal axons of neurons. Protein levels in this microenvironment are influenced not only by anterograde axonal transport from proximal neuronal cell bodies but also by local cells such as Schwann cells, fibroblasts, infiltrating immune cells, and various stem cells [89]. For example, studies have shown elevated transcript or protein levels of CXCL5, CX3 CL1, and XCL1 in neuronal cell bodies following nerve injury [36, 38]. However, owing to a lack of expression data for these proteins in local cells and in the overall injured tissue, comprehensively assessing their expression within the injury microenvironment is currently challenging.

**Fig. 2:**
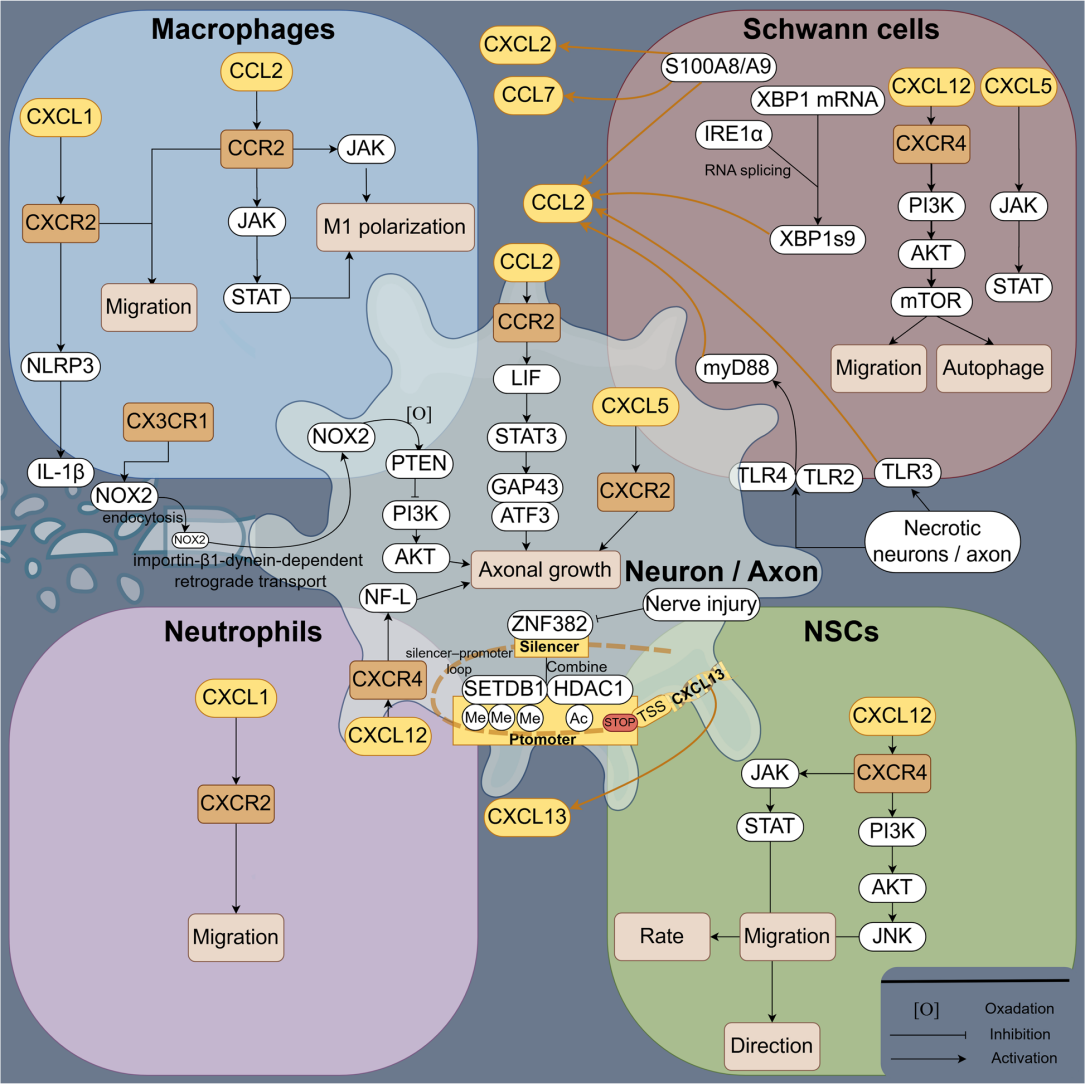
Expression and mechanism of action of chemokine family members in PNI. Macrophages: In macrophages, CXCL1/CXCR2 promotes the expression of NLRP3, thereby facilitating the release of IL- 1β. CCL2/CCR2 activates signaling pathways such as the JAK-STAT and Ras pathways to promote M1 polarization. The activation of CX3 CR1 results in the release of NOX2, which enters damaged axons via endocytosis and is transported to the cell body via retrograde transport through an importin-β1-dynein-dependent mechanism. NOX2 oxidizes PTEN, leading to its inactivation and subsequent activation of the PI3 K‒Akt signaling pathway, thus promoting nerve regeneration

Chemokine receptors are widely expressed across various cell types, including neurons/axons, Schwann cells, fibroblasts, immune cells, and tissue-derived stem cells. This broad distribution allows chemokines to influence multiple aspects of the nerve repair process through both direct and indirect mechanisms. As illustrated in Fig. 2, the core functions of chemokine family members focus on neurotrophic support and inflammation regulation, including the secretion of inflammatory factors and chemotactic activity.

Schwann cells: In Schwann cells, homogenates of necrotic neurons or nerve tissues activate TLR2, TLR3, and TLR4 to release CCL2, with TLR2 and TLR4 promoting CCL2 expression via a MyD88-dependent pathway. IRE1α catalyzes splicing of the Xbp1 mRNA to generate the active transcription factor XBP1 s, increasing CCL2 expression. S100 A8/A9 upregulates CCL2, CCL7, and CXCL2 expression. CXCL5 activates the JAK-STAT pathway. CXCL12 activates the PI3 K/AKT/mTOR pathway, enhancing Schwann cell migration and autophagy. Neutrophils: In neutrophils, CXCL1/CXCR2 mediates neutrophil recruitment. Neurons/axons: In neurons, ZNF382 interacts with the distal silencer region of the CXCL13 gene and forms a complex with HDAC1 and SETDB1 in the promoter region. This complex occupies the promoter and 5′-UTR regions, creating a silencer–promoter loop that suppresses CXCL13 transcription in normal DRG neurons. Neural injury reduces ZNF382 expression, leading to the release of CXCL13. CXCL5/CXCR2 promotes axonal growth. CXCL12/CXCR4 increases NF-L expression, facilitating nerve growth. CCL2 directly promotes neuronal regeneration via a STAT3-dependent mechanism. CCL2 overexpression selectively increases LIF mRNA levels and activates STAT3 to promote nerve growth. Additionally, CCL2 increases the expression of GAP43 and ATF3 in DRG neurons, significantly enhancing nerve regeneration. NSCs: In NSCs, CXCL12/CXCR4 regulates migration speed via the ERK1/2-p38MAPK pathway and directs migration through the AKT‒JNK pathway.

A classical function of chemokines is regulation of the inflammatory response, which encompasses the release of inflammatory factors and the recruitment of immune cells—an essential aspect of nerve repair. After PNI, macrophages and neutrophils recruited by chemokines play central roles in Wallerian degeneration by clearing myelin debris and creating an environment conducive to subsequent nerve regeneration [21, 76]. However, as Wallerian degeneration concludes and the proximal and distal axons begin to reconnect—signaling the late stage of nerve repair—excessive inflammatory responses may inhibit the remyelination capacity of repair Schwann cells and impede the transition of macrophages from the M1 to M2 phenotype [90]. This dual role of inflammation may explain why studies of some anti-inflammatory agents that do not specifically target chemokines reported significant therapeutic effects even when chemokine levels were reduced. Therefore, we propose that the therapeutic window for chemokines is likely concentrated during the early stages of acute injury, whereas sustained high levels of chemokines during the later stages of injury may be detrimental to tissue repair.

Certain models of chronic neuronal sensitization induced by persistent injury may not be suitable for evaluating the role of chemokines in nerve repair. First, neuronal sensitization and nerve regeneration are two distinct processes. Although the proinflammatory effects of chemokines may lead to neuronal sensitization and contribute to pain hypersensitivity, this does not necessarily impede nerve repair and regeneration. For example, CXCL5 and CCL2 have been shown in previous studies to have dual roles—in both promoting neuronal sensitization and facilitating nerve repair [54, 56, 59]. Furthermore, in models such as chronic constriction injury (CCI) [91], spinal nerve ligation (SNL) [92], and chronic compression of the dorsal root ganglion (CCD) [92], the continual presence of injurious factors prevents effective nerve repair. This makes it challenging to accurately assess interventions aimed at promoting nerve regeneration. Consequently, we suggest that using crush or transection models may provide a more direct and observable measure of how interventions affect nerve repair. Additionally, when conducting behavioral assessments, it is important to consider the influence of neuronal sensitization in order to accurately reflect the true progress of nerve recovery.

## Conclusions

In conclusion, numerous chemokines have been reported to promote peripheral nerve repair through direct or indirect mechanisms. Future research should focus on elucidating the precise localization and temporal expression patterns of these chemokines, as well as exploring therapeutic approaches—such as recombinant protein technology, exosome technology, and stem cell therapy—that modulate chemokine levels in tissues to enhance nerve repair. It is also essential to closely consider the timing of chemokine-based therapies and compare their effects during the early and late phases of injury, as well as throughout the entire healing process, to determine the optimal administration window and mitigate potential adverse effects. Moreover, CXCL5, CXCL12, and CCL2 may have a particularly critical role in peripheral nerve repair due to their direct neuroprotective effects, warranting further focused investigation.

## Data Availability

Not applicable.

## Abbreviations

PNI: Peripheral nerve injuries
ES: Electrical stimulation
PNS: Peripheral nervous system
CNS: Central nervous system
rSCs: Repair Schwann cells
DRG: Dorsal root ganglion
ADSCs: Adipose-derived stem cells
ROS: Reactive oxygen species
MPG: Major pelvic ganglion
PRP: Platelet-rich plasma
BCNC: Bilateral cavernous nerve crush
DLSW: Low-energy defocused shock wave
BMSCs: Bone marrow-derived mesenchymal stromal cells
RGCs: Retinal ganglion cells
CCI: Chronic constriction injury
NF-L: Neurofilament light chain neurofilament light chain
MSCs: Mesenchymal stem cells
PCNs: Polyelectrolyte complex nanoparticles
NSCs: Neural stem cells
ac-H3: Acetylation of histone H3
ER: Endoplasmic reticulum
UPR: Unfolded protein response
COS: Chitooligosaccharide
sSiglec-9: Sialic acid-binding immunoglobulin-like lectin-9
SHED-CM: Stem cells from human exfoliated deciduous teeth
NOX2: NADPH oxidase 2
XCL1: Lymphotactin-α
XCL2: Lymphotactin-β
CN: Cavernous nerve
CCD: Chronic compression of the dorsal root ganglion
SNL: Spinal nerve ligation
